# Effect of hypoalbuminemia on postoperative pulmonary complications after thoracoscopic anatomical lung resection: a retrospective cohort study

**DOI:** 10.7717/peerj.21456

**Published:** 2026-06-11

**Authors:** Mengmeng Xu, Jie Sun, Menghan Sun

**Affiliations:** 1 Department of Anesthesiology, The First Affiliated Hospital of USTC, Division of Life Science and Medicine, University of Science and Technology of China, Hefei, Anhui Province, China; 2 Department of Anesthesiology, The First Affiliated Hospital of Nanjing Medical University, Nanjing Medical University, Nanjing, Jiangsu Province, China; 3 Department of Anesthesiology, Zhongda Hospital, School of Medicine, Southeast University, Nanjing, Jiangsu Province, China

**Keywords:** Postoperative pulmonary complications, Risk factors, Propensity score matching

## Abstract

**Objectives:**

Anatomical segmental resection is a standard treatment for non-small cell lung cancer but frequently entails postoperative pulmonary complications (PPCs). Serum albumin level, as useful assessment of changes in nutritional status, is closely associated with postoperative outcomes. Therefore, we evaluated the impact of preoperative albuminemia level on PPCs in thoracoscopic anatomical lung resection.

**Methods:**

We conducted a retrospective analysis of clinical data from 1,192 patients who underwent elective thoracoscopic anatomical lung resection owing to pulmonary tumors or other diseases at the First Affiliated Hospital of Nanjing Medical University between January 2020 and December 2020. Patients were stratified into a hypoalbuminemia group (<35 g/L) and a normal albumin group (≥35 g/L) based on the lowest albumin concentration in the 48 h before operation. Demographics, perioperative data, and PPCs incidence were compared between groups. Propensity score matching (PSM) was employed to balance baseline characteristics, after which PPCs incidence was recompared. Multivariate regression was performed to assess excess risks for PPCs associated with hypoalbuminemia, adjusted for demographics and clinical variables.

**Results:**

Among the 1,192 patients included, the overall incidence rate of PPCs was 26.7% (319/1,192), with pneumonia being the most common complication (16.0%). Preoperative hypoalbuminemia was present in 224 patients (18.8%). After PSM, the hypoalbuminemia group had a significantly higher incidence of PPCs compared to the normal albumin group (*P* < 0.05). Multivariate logistic regression identified hypoalbuminemia as an independent risk factor for PPCs. Additionally, patients with hypoalbuminemia had a longer postoperative hospital stay.

**Conclusion:**

Preoperative hypoalbuminemia is an independent predictor of PPCs, particularly pneumonia, in patients with normal pulmonary function undergoing thoracoscopic anatomical lung resection.

## Introduction

Video-assisted thoracoscopic surgery (VATS) anatomical lung resection has become a standard procedure for the treatment of lung cancer due to its minimally invasive nature and association with accelerated postoperative recovery (Imperatori et al., 2008; Cao et al., 2025). Despite the widespread adoption of enhanced recovery after surgery (ERAS) protocols and ongoing advancements in surgical techniques, postoperative pulmonary complications (PPCs) remain the most common morbidity following lung cancer surgery, occurring in 12–40% of patients (Kinugasa et al., 2004; Kaufmann & Heinrich, 2021). PPCs are associated with increased in-hospital mortality, higher healthcare costs, prolonged length of stay, and delayed functional recovery (Simonsen et al., 2015; Lugg et al., 2016). The development of PPCs is multifactorial, involving both patient-and procedure-related factors (Simonsen et al., 2015; Miskovic & Lumb, 2017; Haines & Agarwal, 2017). Distinguishing between modifiable and non-modifiable risk factors is essential for the timely identification of high-risk patients and the optimization of perioperative management.

Perioperative albumin levels have been identified as another potential risk factor for postoperative adverse outcomes (Hu et al., 2019; Bohl et al., 2017; Hardt et al., 2017; Im et al., 2019; Bohl et al., 2016; Okada et al., 2018), including PPCs (Qaseem et al., 2006; Arslantas et al., 2015; Peacock et al., 2017; Wu et al., 2019), and one that is potentially modifiable. However, studies investigating the impact of hypoalbuminemia on PPCs following lung resection remain limited, with most focusing on postoperative hypoalbuminemia (Li et al., 2018; Saji et al., 2018). The preoperative identification of high-risk patients is of paramount importance to the development of targeted preventive strategies. Therefore, this study aimed to determine whether preoperative hypoalbuminemia is associated with PPCs in patients with normal pulmonary function undergoing VATS anatomical lung resection.

## Materials & Methods

### Study population

This retrospective study was approved by the Ethics Committee of the First Affiliated Hospital of Nanjing Medical University (Approval No. 2019-SR-234). The requirement for informed consent was waived due to the retrospective design. We included patients with normal lung function who underwent VATS anatomical lung resection at our institution between January 2020 and December 2020.

Inclusion criteria were: (1) undergoing their first VATS lobectomy or segmentectomy; (2) age ≥18 years; (3) American Society of Anesthesiologists (ASA) physical status I–III; (4) patients with normal pulmonary function and without obvious severe liver dysfunction; (5) Cancer (AJCC) tumor-node-metastasis (TNM) staging I to II. Exclusion criteria were: (1) any other surgery in the 3 months prior to VATS; (2) albumin infusion during the perioperative period; (3) signs of active infection or comorbid conditions affecting serum albumin levels; (4) incomplete or inaccessible medical data.

### Data collection

Data included patient characteristics, ASA physical status, smoking history, pre-existing comorbidities, surgical procedures (surgical approach, type of resection, and pathologic diagnosis), surgeons. Preoperative laboratory data (including serum albumin levels) and intraoperative variables (operation time, urine volume, blood loss volume, total infusion volume) were recorded. Hypoalbuminemia was defined as a serum albumin level <35 g/L (Bohl et al., 2016). Perioperative care followed standardized protocols for all patients.

### Definition of outcomes

The primary outcome was the incidence of in-hospital PPCs, including pneumonia, prolonged air leak, atelectasis, pleural effusion, acute respiratory distress syndrome (ARDS), pulmonary embolism, and reintubation.

Pneumonia was defined as a new pulmonary infiltrate with associated increase in white blood cells and fever. Prolonged air leak was accepted as leak >7 days. Atelectasis was defined as an area of no ventilation or collapse identified on chest radiograph. Pleural effusion was defined as chest radiograph with blunting of costophrenic angle, loss of sharp silhouette of the ipsilateral hemidiaphragm in upright position, displacement of adjacent anatomical structures, or (in supine position) hazy opacity in one hemithorax with preserved vascular shadows. ARDS was defined as acute onset of hypoxemia with abnormal oxygenation ratios (arterial partial pressure of oxygen to fraction of inspired oxygen: ARDS < 300) and radiologic infiltrates characteristic of pulmonary edema. The diagnostic criteria of PPCs used in this study were based on the National Surgical Quality Improvement Program, the Society of Thoracic Surgeons, and the European Society of Thoracic Surgeons definitions for postoperative pulmonary complications (Fernandez et al., 2015).

### Statistical analysis

Continuous variables are presented as mean ± standard deviation or median (interquartile range) based on distribution normality and were compared using unpaired t-tests or Mann–Whitney U tests. Categorical variables are expressed as percentages and compared using Pearson’s chi-square test or Fisher’s exact test, as appropriate. To mitigate selection bias, propensity score matching (PSM) was performed with the hypoalbuminemia group as the treatment group. Matching variables included age, sex, BMI, ASA grade, smoking history, and previous comorbidities. A 1:4 nearest-neighbor matching algorithm with a caliper width of 0.1 standard deviations was used. Univariate and multivariate logistic regression analyses were conducted to identify significant predictors of PPCs. Variables with *P* < 0.1 in univariate analysis and known prognostic factors were included in the multivariate model. Results are presented as odds ratios (OR) with 95% confidence intervals (CIs). All tests were two-tailed, with *P* < 0.05 considered statistically significant. Analyses were performed using SPSS 22.0 (IBM Corp., Armonk, NY, USA) and R version 3.5.3.

## Results

### Patient characteristics

A total of 1,192 patients meeting the inclusion criteria were analyzed. The overall incidence of PPCs was 26.7% (*n* = 319). Pneumonia was the most common type of PPC (*n* = 191, 16.0%), followed by pleural effusion (*n* = 95, 8.0%); prolonged air leak (*n* = 52, 4.3%), and atelectasis (*n* = 17, 1.4%). No cases of ARDS, pulmonary embolism, reintubation, or in-hospital mortality occurred. Preoperative hypoalbuminemia was present in 224 patients (18.8%), while 968 patients had normal serum albumin levels.

### Propensity score matching

Patient demographics and clinical characteristics before and after propensity score matching are shown in Table 1. Before matching, the hypoalbuminemia group was significantly older, had a higher proportion of males, lower BMI, and higher rates of smoking history compared to the normal albumin group (all *P* < 0.05). After PSM, these differences were balanced (all standardized differences < 0.1).

**Table 1:** Characteristics before and after propensity score matching (PSM).

Characteristics	Before PSM		*P* value	After PSM		*P* value
	Normal albumin group (*n* = 968)	Hypoalbuminemia group (*n* = 224)		Normal albumin group (*n* = 698)	Hypoalbuminemia group (*n* = 215)	
**Age** (years)	55.5 ± 10.4	60.0 ± 9.6	0.001	57.6 ± 9.6	59.6 ± 9.5	0.070
**Male**	382 (39.5%)	111 (49.6%)	0.006	402 (57.6%)	113 (52.6%)	0.193
**BMI** (kg/m^2^)	23.6 ± 3.0	22.9 ± 3.2	0.003	23.2 ± 2.9	23.0 ± 3.2	0.381
**ASA**			0.001			0.080
I	123 (12.7%)	21 (9.4%)		71 (10.2%)	21 (9.8%)	
II	770 (79.5%)	165 (73.7%)		561 (80.4%)	162 (75.3%)	
III	75 (7.7%)	38 (17.0%)		66 (9.5%)	32 (14.9%)	
**Smoking**	274 (28.3%)	82 (36.6%)	0.014	216 (30.9%)	76 (35.3%)	0.226
**Hypertension**	96 (9.9%)	19 (8.5%)	0.512	64 (9.2%)	16 (7.4%)	0.434
**Diabetes** (T1DM or T2DM)	46 (4.8%)	5 (2.2%)	0.093	25 (3.6%)	5 (2.3%)	0.366
**CHD**	25 (2.6%)	5 (2.2%)	0.763	14 (2.0%)	4 (1.9%)	0.893
**Right lung lobe**	566 (58.5%)	137 (61.2%)	0.461	408 (58.5%)	130 (60.5%)	0.600
**Type of anatomical lung resection**			0.001			0.001
Single lobectomy	592 (61.2%)	170 (75.9%)		437 (62.6%)	162 (75.3%)	
Single segmentectomy	251 (25.9%)	44 (19.6%)		165 (23.6%)	43 (20.0%)	
Bilobectomy or combined lobectomy and segmentectomy	125 (25.9%)	10 (4.5%)		96 (13.8%)	10 (4.7%)	
**Pathology**			0.001			0.001
Adenocarcinoma	844 (87.2%)	172 (76.8%)		615 (88.1%)	168 (78.1%)	
Squamous cell carcinoma	26 (2.7%)	18 (8.0%)		22 (3.2%)	17 (7.9%)	
Inflammatory nodule	42 (4.3%)	19 (8.5%)		26 (3.7%)	18 (8.4%)	
Other	56 (5.8%)	15 (6.7%)		35 (5.0%)	12 (5.6%)	
**S** **urgon**			0.051			0.076
1	314 (32.4%)	57 (25.4%)		231 (33.1%)	54 (25.1%)	
2	139 (14.4%)	30 (13.4%)		94 (13.5%)	29 (13.5%)	
3	308 (31.8%)	92 (41.4%)		226 (32.4%)	88 (40.9%)	
4	207 (21.4%)	45 (20.1%)		147 (21.1%)	44 (20.5%)	
**Length of operation** (h)	2.2 ± 0.6	2.4 ± 0.8	0.009	2.2 ± 0.7	2.4 ± 0.8	0.009
**Intraoperative input fluid** (mL)	1,450 (1,200, 1,600)	1,500 (1,300, 1,650)	0.042	1,450 (1,200, 1,650)	1,500 (1,300, 1,630)	0.040
**Colloid** (mL)	500 (0, 500)	500 (438, 500)	0.016	500 (0, 500)	500 (350, 500)	0.044
**Intraoperative bleeding** (mL)	50 (20, 100)	50 (20, 100)	0.234	50 (20, 100)	50 (20, 100)	0.009
**Intraoperative urine output** (mL)	300 (200, 500)	300 (200, 500)	0.567	300 (200, 500)	300 (200, 500)	0.664
**PPCs**	239 (24.7%)	80 (35.7%)	0.001	179 (25.6%)	78 (36.3%)	0.002
**Pneumonia**	133 (13.7%)	58 (25.9%)	0.001	100 (14.3%)	57 (26.5%)	0.001
**A** **ir leakage**	41 (4.2%)	11 (4.9%)	0.656	29 (4.2%)	11 (5.1%)	0.547
**Pleural effusion**	82 (8.5%)	13 (5.8%)	0.184	63 (9.0%)	12 (5.6%)	0.108
**Atelectasis**	12 (1.2%)	5 (2.2%)	0.259	8 (1.1%)	5 (2.3%)	0.202
**Length of stay** (d)	5 (4,7)	5 (4,6)	0.001	5 (4,6)	5 (4,7)	0.001

**Notes.**

Values are presented as mean ± SD, n, or n (%).

BMI body mass index ASA American Society of Anesthesiologists CHD coronary heart disease PPCs postoperative pulmonary complications

Diabetic patients referred to those diagnosed with type 1 or type 2 diabetes mellitus.

Among the 215 matched hypoalbuminemia patients, 78 (36.3%) developed PPCs and 57 (26.5%) developed pneumonia. In the matched normal albumin group (*n* = 698), 179 (25.6%) developed PPCs and 100 (14.3%) developed pneumonia. The differences in PPCs and pneumonia incidence between groups were statistically significant (*P* = 0.02 and *P* = 0.001, respectively). Hospital stay was longer in the hypoalbuminemia group (*P* = 0.001). No significant differences were observed in the incidence of prolonged air leak, pleural effusion, or atelectasis between groups after matching (Table 1).

Given the potential confounding bias from surgical procedure type on the outcomes, we conducted a subgroup analysis by type of anatomical lung resection following PSM. The results showed that the association between preoperative hypoalbuminemia and PPCs, particularly pneumonia, was only observed in patients undergoing single lobectomy (*P* = 0.001) (Table S1).

### Multivariate logistic regression

**Figure 1:**
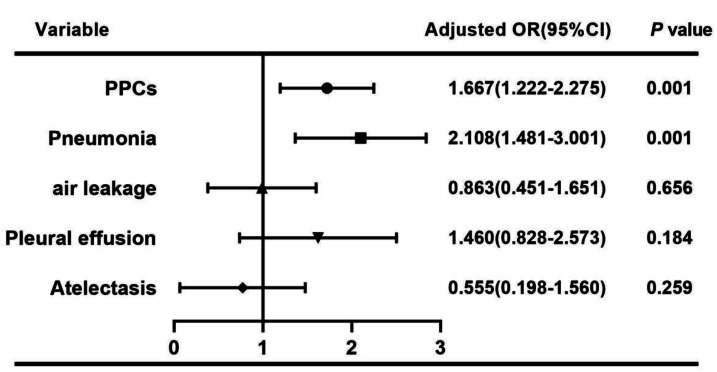
Forest map and logistic regression results of the influence of hypoalbuminemia on various pulmonary complications.

Univariate analysis comparing patients with and without PPCs (Table 2) identified significant differences in serum albumin levels, surgeon, surgical lobe, intraoperative bleeding, and operative duration. Multivariate logistic regression confirmed that hypoalbuminemia was an independent risk factor for PPCs (OR: 1.667, 95% CI [1.222–2.275]; *P* = 0.001) and specifically for pneumonia (OR: 2.108, 95% CI [1.481–3.001]; *P* = 0.001). Hypoalbuminemia was not an independent risk factor for prolonged air leak, pleural effusion, or atelectasis (Fig. 1).

**Table 2:** Intraoperative and postoperative factors of patients with or without postoperative pulmonary complications by univariate analysis (*n* = 1,192).

	Univariate analysis			
Characteristics	Entire (*n* = 1,192)		PPCs group (*n* = 319)		No PPCs group (*n* = 873)	*P*-value
**Age** (years)	6.4 ± 10.4	56.4 ± 10.5	56.5 ± 10.0	0.825
**Male**	493 (41.4%)	138 (43.3%)	355 (40.7%)	0.420
**BMI** (kg/m^2^)	23.5 ± 3.1	23.6 ± 3.2	23.5 ± 3.0	0.546
**ASA**				0.222
I	144 (12.1%)	38 (11.9%)	106 (12.1%)	
II	935 (78.4%)	243 (76.2%)	692 (79.3%)	
III	113 (9.5%)	38 (11.9%)	75 (8.6%)	
**Smoking**	356 (29.9%)	106 (33.2%)	250 (28.6%)	0.125
**Hypertension**	115 (9.6%)	33 (10.3%)	82 (9.4%)	0.622
**Diabetes** (T1DM or T2DM)	1 (4.3%)	11 (3.4%)	40 (4.6%)	0.392
**CHD**	30 (2.5%)	7 (2.2%)	23 (2.6%)	0.667
**Hypoalbumiemia**	224 (18.8%)	80 (25.1%)	144 (16.5%)	0.001
**Right lung lobe**	703 (59.0%)	201 (63.0%)	502 (57.6%)	0.095
**Type of anatomical lung resection**				0.525
Single lobectomy	760 (63.8%)	202 (63.3%)	558 (64.1%)	
Single segmentectomy	295 (24.7%)	85 (26.6%)	210 (24.1%)	
Bilobectomy or combined lobectomy and segmentectomy	135 (11.3%)	32 (10.0%)	103 (11.8%)	
**Pathology**				0.097
Adenocarcinoma	1,014 (85.1%)	261 (81.8%)	753 (86.5%)	
Squamous cell carcinoma	44 (3.7%)	16 (5.0%)	28 (3.2%)	
Inflammatory nodule	61 (5.1%)	23 (7.2%)	38 (4.4%)	
other	71 (6.0%)	19 (6.0%)	52 (6.0%)	
**Surgeon**				0.077
1	371 (31.1%)	81 (25.4%)	290 (33.2%)	
2	169 (14.2%)	47 (14.7%)	122 (14.0%)	
3	400 (33.6%)	118 (37.0%)	282 (32.3%)	
4	252 (21.1%)	73 (22.9%)	179 (20.5%)	
**Length of operation** (h)	2.3 ± 0.7	2.3 ± 0.7	2.2 ± 0.7	0.023
≥2 h	733 (61.5%)	211 (66.1%)	522 (59.8%)	0.046
**Intraoperative input fluid** (mL)	1,500 (1,200, 1,600)	1,450 (1,200, 1,600)	1,500 (1,250, 1,610)		0.335
**Colloid** (mL)	00 (0, 500)	500 (175, 500)	500 (0, 500)	0.243
**Intraoperative bleeding** (mL)	0 (20, 100)	50 (30, 120)	50 (20, 100)	0.010
**Intraoperative urine output** (mL)	300 (200, 500)	300 (200, 490)	300 (200, 500)	0.848
**Length of stay** (d)	(4,6)	5 (4,7)	5 (4,6)	0.001

**Notes.**

Values are presented as mean ± SD, n, or n (%).

BMI body mass index ASA American Society of Anesthesiologists CHD coronary heart disease PPCs postoperative pulmonary complications

Diabetic patients referred to those diagnosed with type 1 or type 2 diabetes mellitus.

## Discussion

In this study, the incidence of pulmonary complications in patients with normal preoperative lung function who underwent VATS anatomical lung resection was 26.7%. This finding is presumably attributable to the enrollment of patients with ASA physical status I to III, which may explain the absence of postoperative severe hypoxemia, respiratory failure, or pulmonary embolism.

A high prevalence of preoperative hypoalbuminemia (serum albumin < 35 g/L) was identified in the cohort, accounting for 18.8% of patients. Hypoalbuminemia can induce edema in tissues and organs, delay physiological recovery, compromise anti-infective capacity, and increase morbidity and mortality (Hennessey et al., 2010). Existing studies have reported that hypoalbuminemia is a risk factor for PPCs (Hu et al., 2019; Bohl et al., 2016). In a large retrospective observational study, Bohl et al. (2016) found that hypoalbuminemia increases the risk of PPCs following orthopedic surgery by 1.5-fold, even after adjusting for patient- and surgery-related factors. Postoperative pneumonia and pulmonary embolism following colorectal surgery have also been associated with hypoalbuminemia (Hu et al., 2019). However, baseline preoperative pulmonary function was not taken into account in most prior studies.

This study included 1,192 patients with normal pulmonary function undergoing their first thoracoscopic lung resection. After incorporating potential perioperative influencing factors into regression analysis, we found that preoperative hypoalbuminemia was an independent risk factor for PPCs, particularly pneumonia, but not for atelectasis, prolonged air leak, or pleural effusion. Furthermore, this result was supported by PSM analysis, which showed that the incidence of PPCs, and of pneumonia in particular, was significantly higher in the hypoalbuminemia group compared to the normal albumin level group. A study demonstrated that patients with *a* ≥ 14.97% decrease in albumin levels were at higher risk of developing PPCs after VATS anatomical lung cancer resection (Li et al., 2018). They also identified preoperative pulmonary function as an independent risk factor for PPCs, which verified the effectiveness of our study design that controlled for the influence of pulmonary function on postoperative complications, to some extent. However, calculating the ΔALB (changes in albumin level) postoperatively is not entirely convenient for clinical application. In contrast, preoperative albumin levels offer a more straightforward means of identifying high-risk patients, thereby enabling the implementation of proactive perioperative protective measures to reduce the incidence of PPCs. Hypoalbuminemia was reportedly associated with prolonged air leak (Okada et al., 2017; Isowa et al., 2002). Another study suggested low preoperative serum albumin can serve as a significant risk factor for postoperative bronchopleural fistula in patients undergoing pulmonary resections (Zhang et al., 2019). However, this association was not found in our study. This may be because these complications are more directly related to surgical technique or their incidence was too low in our cohort to detect a statistically significant relationship.

The potential mechanisms by which hypoalbuminemia may contribute to the development of PPCs include the following aspects: (1) Serum albumin serves not only as a plasma buffer to maintain physiological pH but also as a biomarker reflecting systemic physiological status. Clinical conditions such as malnutrition, hepatic insufficiency, and hemodilution can significantly reduce serum albumin levels, which in turn may adversely affect postoperative outcomes in surgical patients (Bohl et al., 2016; Qaseem et al., 2006; Saji et al., 2018). (2) Serum albumin regulates the function of various proteins and biomolecules through specific binding and transport mechanisms. Decreased serum albumin levels can inhibit wound healing by impairing fibroblast proliferation and collagen synthesis. Maintaining endothelial barrier integrity also requires continuous albumin synthesis and repair (Alphonsus & Rodseth, 2014). Hypoalbuminemia can damage the endothelial glycocalyx, leading to increased vascular permeability, extravasation of albumin and fluid into the interstitium, and consequent impairment of tissue perfusion (Kim et al., 2016). (3) As the primary extracellular antioxidant in the body, albumin scavenges more than 70% of circulating free radicals. A reduction in serum albumin levels delays free radical clearance, thereby exacerbating the perioperative systemic inflammatory response syndrome (SIRS) (Gatta, Verardo & Bolognesi, 2012; Chen et al., 2015). (4) Albumin levels reflect systemic inflammation and oxidative stress, while persistent chronic inflammation is closely related to the development and progression of idiopathic pulmonary fibrosis (IPF) (Flaherty et al., 2001). Serum albumin can also combine with various substances, transport them, and affect the function of binding proteins such as thioredoxin, which requires albumin binding to more effectively inhibit pulmonary fibrosis (Tanaka et al., 2013). This finding suggests that clinicians should pay attention not only to the impact of albumin on short-term postoperative complications but also to its relationship with long-term prognosis.

Does perioperative albumin supplementation reduce the incidence of PPCs or other systemic complications? Lee et al. (2016) demonstrated that perioperative albumin supplementation may improve renal function after off-pump cardiac surgery. However, the clinical value of exogenous albumin supplementation remains controversial. While some studies suggest that albumin supplementation can enhance organ function in critically ill patients with hypoalbuminemia (Dubois et al., 2006), a growing body of evidence indicates potential adverse effects, including altered coagulation profiles, renal impairment, reduced myocardial contractility, and immunosuppression (Pulimood & Park, 2000). Kim et al. (2017) concluded that intravenous albumin infusion aimed solely at correcting preoperative hypoalbuminemia has little impact on improving postoperative outcomes. Some guidelines recommend albumin supplementation only in major surgeries such as hepatectomy or extensive bowel resection, and when serum albumin levels fall below 20 g/L (Liumbruno et al., 2009). Currently, routine albumin supplementation is not recommended for mild hypoalbuminemia; instead, efforts should focus on addressing the underlying causes, such as improving nutritional status (Bohl et al., 2016).

The present analysis has several limitations. First, as a single-center retrospective analysis utilising hospital electronic medical record data, there are inherent limitations to this work including selection and reporting biases, which limit the generalizability of the findings. Second, owing to the retrospective study design, we cannot completely exclude the possibility of unmeasured or unknown confounding factors (such as frailty, ventilation strategy, hemodynamic data, postoperative pain management strategies, *etc.*) that may contribute to the associations observed in this study. Third, we performed propensity score matching to adjust for potential confounding factors, including preoperative comorbidities. After matching, all standardized mean differences were below 0.1, demonstrating adequate balance between the two groups. Although the sample size was reduced relative to the original dataset, which exhibited substantial confounding bias prior to matching, the balance between the groups was markedly improved. Finally, we only investigated the relationship between hypoalbuminemia and short-term PPCs; the impact of hypoalbuminemia on other postoperative complications and long-term adverse events remains unknown. New studies and data are required to elaborate the optimal perioperative serum albumin level for improving patient outcomes.

## Conclusions

This study demonstrates that preoperative hypoalbuminemia is an independent predictor of PPCs, particularly pneumonia, in patients with normal pulmonary function undergoing thoracoscopic anatomical lung resection. Preoperative albumin levels should be incorporated into comprehensive risk assessment by surgeons and anesthesiologists. Targeted interventions to correct hypoalbuminemia, along with optimized perioperative management, may improve postoperative outcomes. Further prospective studies are needed to confirm these findings and explore potential preventive strategies.

##  Supplemental Information

10.7717/peerj.21456/supp-1 Supplemental Information 1 STROBE checklist

10.7717/peerj.21456/supp-2 Supplemental Information 2 Raw data

10.7717/peerj.21456/supp-3 Supplemental Information 3 Codebook for categorical data

10.7717/peerj.21456/supp-4 Supplemental Information 4 Subgroup analysis by type of anatomical lung resection following the propensity score matching

## Additional Information and Declarations

### Data Availability

The following information was supplied regarding data availability: The raw data are available in the Supplemental Files.
