# Supplementary material for: Effect of hypoalbuminemia on postoperative pulmonary complications after thoracoscopic anatomical lung resection: a retrospective cohort study
Source: PeerJ. 2026 Jun 11;14:e21456. doi: 10.7717/peerj.21456 (PMC13264973; doi:10.7717/peerj.21456)
Supplement: Supplemental Information 1 [file peerj-14-21456-s001.docx]

STROBE Statement—checklist of items that should be included in reports of observational studies

|  | Item No. | Recommendation | Page  No. | Relevant text from manuscript |
| --- | --- | --- | --- | --- |
| **Title and abstract** | 1 | (*a*) Indicate the study’s design with a commonly used term in the title or the abstract | 1 | A propensity score-matched analysis. |
|  |  | (*b*) Provide in the abstract an informative and balanced summary of what was done and what was found | 1-2 | We retrospectively analyzed 1192 cases and found that preoperative hypoalbuminemia was associated with an increased risk of postoperative pulmonary complications in patients after video-assisted thoracic surgery. |
| Introduction | | | |  |
| Background/rationale | 2 | Explain the scientific background and rationale for the investigation being reported | 2 | Despite the improvement in surgical techniques, postoperative pulmonary complications (PPCs) still are the most common complications after lung cancer surgery, and occur in 12%–40% of patients after surgical lung resection, and many perioperative-associated factors for PPCs after lung resection are not being researched and understood. |
| Objectives | 3 | State specific objectives, including any prespecified hypotheses | 2 | We needed to clarify whether perioperative hypoalbuminemia is related to PPCs in patients with normal pulmonary function after VATS. |
| Methods | | | |  |
| Study design | 4 | Present key elements of study design early in the paper | 2 | This study included patients with normal lung function who underwent VATS at our hospital. |
| Setting | 5 | Describe the setting, locations, and relevant dates, including periods of recruitment, exposure, follow-up, and data collection | 2 | This study included patients with normal lung function who underwent VATS at our hospital between January 2020 and December 2020. |
| Participants | 6 | (*a*) *Cohort study*—Give the eligibility criteria, and the sources and methods of selection of participants. Describe methods of follow-up  *Case-control study*—Give the eligibility criteria, and the sources and methods of case ascertainment and control selection. Give the rationale for the choice of cases and controls  *Cross-sectional study*—Give the eligibility criteria, and the sources and methods of selection of participants | 2-3 | The inclusion criteria were as follows: (1) first time undergoing VATS; (2) age ≥18 years; (3) American Society of Anesthesiologists Physical Status classification I to III; and (4) patients with normal pulmonary function and without obvious severe liver dysfunction. (5) Cancer (AJCC) tumor-node-metastasis (TNM) staging I to II. The exclusion criteria were as follows: (1) any other surgery in the 3 months prior to VATS; (2) albumin transfusion during the perioperative period; (3) patients with signs of infection and those combined with disease which may affect the serum albumin; and (4) incomplete or inaccessible data. |
|  |  | (*b*) *Cohort study*—For matched studies, give matching criteria and number of exposed and unexposed  *Case-control study*—For matched studies, give matching criteria and the number of controls per case | 3 | To balance the baseline of patients, propensity score matching was performed, and the hypoalbuminemia group was considered the treatment group, and the variables were age, sex, smoking history, BMI, pathology, surgeons, intraoperative fluid infusion, and surgical duration. The normal albumin group and hypoalbuminemia group pairs with the nearest propensity score were matched 1:4 with a caliper width of 0.1 for standard deviation. |
| Variables | 7 | Clearly define all outcomes, exposures, predictors, potential confounders, and effect modifiers. Give diagnostic criteria, if applicable | 3 | The primary outcome of our study was the incidence of in-hospital PPCs occurrence. The PPCs included pneumonia, air leak, atelectasis, acute respiratory distress syndrome (ARDS), pulmonary embolism, and re-intubation.^7, 14, 15^ The criteria used to define PPCs were according to the joint definitions of the National Surgical Quality Improvement Program, the Society of Thoracic Surgeons, and the European Society of Thoracic Surgeons. Hypoalbuminemia was defined as a serum albumin level <35 g/L.^13^ |
| Data sources/ measurement | 8* | For each variable of interest, give sources of data and details of methods of assessment (measurement). Describe comparability of assessment methods if there is more than one group | 3 | The medical data of patients including patients’ demographics, preoperative investigations, and perioperative variables were collected and reviewed. Preoperative characteristics evaluated included age, sex, BMI (kg/m2), American Society of Anesthesiologists (ASA) grade, and risk factors (smoking history, diabetes mellitus, hypertension, cardiovascular disease). |
| Bias | 9 | Describe any efforts to address potential sources of bias | 3 | To balance the baseline of patients, propensity score matching was performed |
| Study size | 10 | Explain how the study size was arrived at | NA |  |

Continued on next page

| Quantitative variables | 11 | Explain how quantitative variables were handled in the analyses. If applicable, describe which groupings were chosen and why | 3 | Continuous variables were described using mean values with their standard deviation or as medians with interquartile range according to the normality of their distribution and were compared using an unpaired t-test or Mann–Whitney U test as appropriate. |
| --- | --- | --- | --- | --- |
| Statistical methods | 12 | (*a*) Describe all statistical methods, including those used to control for confounding | 3 | Univariable and logistic regression were used to identify the significant prognosis predictors. Predictors (P<0.1) of the univariable analysis and known prognostic factors were incorporated into a multivariable analysis. Results of the univariable and multivariable analyses were presented as odds ratios (OR) and 95% confidence intervals (CIs). All significance tests were two tailed with P<0.05 considered statistically significant. |
|  |  | (*b*) Describe any methods used to examine subgroups and interactions | NA |  |
|  |  | (*c*) Explain how missing data were addressed | NA |  |
|  |  | (*d*) *Cohort study*—If applicable, explain how loss to follow-up was addressed  *Case-control study*—If applicable, explain how matching of cases and controls was addressed  *Cross-sectional study*—If applicable, describe analytical methods taking account of sampling strategy | NA |  |
|  |  | (*e*) Describe any sensitivity analyses | NA |  |
| Results | | | | |
| Participants | 13* | (a) Report numbers of individuals at each stage of study—eg numbers potentially eligible, examined for eligibility, confirmed eligible, included in the study, completing follow-up, and analysed | 4 | Based on the inclusion and exclusion criteria, A total of 1,192 patients who underwent VATS anatomical resection for lung cancer were identified for eligibility this study. |
|  |  | (b) Give reasons for non-participation at each stage | NA |  |
|  |  | (c) Consider use of a flow diagram | NA |  |
| Descriptive data | 14* | (a) Give characteristics of study participants (eg demographic, clinical, social) and information on exposures and potential confounders | Table 1 |  |
|  |  | (b) Indicate number of participants with missing data for each variable of interest | NA |  |
|  |  | (c) *Cohort study*—Summarise follow-up time (eg, average and total amount) | 3 | In-hospital |
| Outcome data | 15* | *Cohort study*—Report numbers of outcome events or summary measures over time | 4 | The incidence of PPCs was 26.7% (n=319) |
|  |  | *Case-control study—*Report numbers in each exposure category, or summary measures of exposure | NA |  |
|  |  | *Cross-sectional study—*Report numbers of outcome events or summary measures | NA |  |
| Main results | 16 | (*a*) Give unadjusted estimates and, if applicable, confounder-adjusted estimates and their precision (eg, 95% confidence interval). Make clear which confounders were adjusted for and why they were included | 4-5 | Binary logistic regression analysis showed that hypoalbuminemia was one of the independent risk factors of PPCs after thoracoscopic anatomical pneumonectomy (adjusted rate ratio, 1.667 [95% CI: 1.222–2.275]; P=0.001). |
|  |  | (*b*) Report category boundaries when continuous variables were categorized | NA |  |
|  |  | (*c*) If relevant, consider translating estimates of relative risk into absolute risk for a meaningful time period | NA |  |

Continued on next page

| Other analyses | 17 | Report other analyses done—eg analyses of subgroups and interactions, and sensitivity analyses | 4-5 | The single factor and multi factor analysis of various pulmonary complications showed that hypoalbuminemia was an independent risk factor of pneumonia (adjusted rate ratio, 2.108 [95% CI: 1.481–3.001]; P=0.001), but not an independent risk factor of prolonged air leakage, pleural effusion, and atelectasis. |
| --- | --- | --- | --- | --- |
| Discussion | | | | |
| Key results | 18 | Summarise key results with reference to study objectives | 5 | In this study, we reported a PPC frequency of 26.7% subjects with normal pulmonary function after thoracoscopic anatomical pneumonectomy, which is still associated with significantly poor short-term outcomes. After matching, pneumonia was still associated with hypoalbuminemia. Our data showed that the risk of PPCs and pneumonia in patients with hypoalbuminemia were higher than in patients with normal serum albumin. |
| Limitations | 19 | Discuss limitations of the study, taking into account sources of potential bias or imprecision. Discuss both direction and magnitude of any potential bias | 6 | This is a single center retrospective analysis, and all patient samples were required to have normal lung function for inclusion. Thus, the likely single center bias and recall bias limit the clinical application of the study results. Second, the primary endpoint of this study was the occurrence of PPCs, and other perioperative morbidities and mortality rate were not explored. In addition, the detailed information of intraoperative hemodynamics and postoperative pain and its treatment were not considered given the retrospective design of the study. |
| Interpretation | 20 | Give a cautious overall interpretation of results considering objectives, limitations, multiplicity of analyses, results from similar studies, and other relevant evidence | 6 | The current study revealed that preoperative hypoalbuminemia predicted pulmonary complications in patients with normal pulmonary function and undergoing thoracoscopic pneumonectomy for the first time. Surgeons are advised to pay attention to preoperative hypoalbuminemia to detect potential pulmonary complications after lung cancer anatomical resection. Appropriate intervention and comprehensive evaluation may improve the postoperative outcome of patients. |
| Generalisability | 21 | Discuss the generalisability (external validity) of the study results | 6 | The likely single center bias and recall bias limit the clinical application of the study results. Therefore, further evidence from future prospective studies is needed to validate our results. |
| Other information | |  | | |
| Funding | 22 | Give the source of funding and the role of the funders for the present study and, if applicable, for the original study on which the present article is based | NA |  |
