# Supplementary material for: Effect of hypoalbuminemia on postoperative pulmonary complications after thoracoscopic anatomical lung resection: a retrospective cohort study
Source: PeerJ. 2026 Jun 11;14:e21456. doi: 10.7717/peerj.21456 (PMC13264973; doi:10.7717/peerj.21456)
Supplement: Supplemental Information 3 [file peerj-14-21456-s003.docx]

| **Variable** | **Assignment method** |
| --- | --- |
| Gender | Male=1, female=2 |
| Smoking | Yes=1, No=0 |
| ASA | I=1, II=2, III=3 |
| Surgical site | Left=1, Right=2 |
| Pathological | Adenocarcinoma=1, Squamous cell carcinoma=2,  Inflammatory nodule=3, Other=4 |
| Type of anatomical lung resection | Single lobectomy=1, Single segmentectomy=2,  Bilobectomy or combined lobectomy and segmentectomy=3 |
| Complications | Yes=1, No=0 |
