# Supplementary material for: Effect of hypoalbuminemia on postoperative pulmonary complications after thoracoscopic anatomical lung resection: a retrospective cohort study
Source: PeerJ. 2026 Jun 11;14:e21456. doi: 10.7717/peerj.21456 (PMC13264973; doi:10.7717/peerj.21456)
Supplement: Supplemental Information 4 [file peerj-14-21456-s004.docx]

|  | Single lobectomy | | | Single segmentectomy | | | Bilobectomy or combined  lobectomy and segmentectomy | | |
| --- | --- | --- | --- | --- | --- | --- | --- | --- | --- |
|  | Normal albumin group | Hypoalbuminemia group | P value | Normal albumin group | Hypoalbuminemia group | P value | Normal albumin group | Hypoalbuminemia group | P value |
| PPCs | 106(24%) | 63(39%) | **0.001** | 50(30%) | 14(32%) | 0.775 | 23(24%) | 1(10%) | 0.316 |
| Pneumonia | 60(14%) | 45(28%) | **0.001** | 29(18%) | 11(26%) | 0.235 | 11(12%) | 1(10%) | 0.890 |
| Air leakage | 16(3%) | 11(7%) | 0.101 | 7(4%) | 0(0%) | 0.169 | 6(6%) | 0(0%) | 0.416 |
| Pleural effusion | 38(9%) | 8(5%) | 0.125 | 17(10%) | 4(9%) | 0.846 | 8(8%) | 0(0%) | 0.342 |
| Atelectasis | 7(2%) | 5(3%) | 0.249 | 0(0%) | 0(0%) | 0.746 | 1(1%) | 0(0%) | 0.202 |

**Supplement Table 1: Subgroup analysis by type of anatomical lung resection**

**following the propensity score matching**

|  |
| --- |
